# Running Distance Is Associated With Components of Total Energy Expenditure in Amenorrhoeic and Non‐Amenorrhoeic Endurance Runners

**DOI:** 10.1111/sms.70362

**Published:** 2026-08-31

**Authors:** Akiko Uchizawa, Emi Kondo, Yasushi Enomoto, Katsuhiko Yajima, Shinsuke Tamai, Koichi Watanabe, Ai Ito, Miki Kosugi, Yasuki Higaki, Hiroyuki Sagayama

**Affiliations:** ^1^ Department of Sports Sciences Japan Institute of Sports Sciences Tokyo Japan; ^2^ Japan Society for the Promotion of Science Tokyo Japan; ^3^ Institute of Health and Sport Sciences University of Tsukuba Ibaraki Japan; ^4^ Advanced Research Initiative for Human High Performance (ARIHHP) University of Tsukuba Ibaraki Japan; ^5^ Department of Sport Sciences Osaka University of Health and Sport Sciences Osaka Japan; ^6^ Faculty of Pharmaceutical Sciences Josai University Saitama Japan; ^7^ Institute of Human Sciences University of Tsukuba Ibaraki Japan; ^8^ Faculty of Sports and Health Science Fukuoka University Fukuoka Japan

## Abstract

Some competitive runners strategically regulate their body weight to optimize race performance by balancing energy intake and expenditure. The constrained total energy expenditure (TEE) model suggests that exercise energy expenditure (EEE) is sustained through compensatory reductions in other components of TEE, including resting metabolic rate (RMR) and sleeping metabolic rate (SMR). However, it remains unclear whether TEE and its components differ between athletes with and without amenorrhoea, or whether the constrained energy model applies to athletes engaged in chronic training. This study compared TEE components—SMR, RMR, non‐exercise activity thermogenesis (NEAT), and EEE—between amenorrhoeic and non‐amenorrhoeic runners and examined their relationship with physical activity, particularly weekly running distance. Twenty female middle‐ and long‐distance runners (10 amenorrhoeic and 10 non‐amenorrhoeic) participated. TEE and body composition were measured under free‐living conditions using the doubly labeled water method, while RMR and SMR were assessed in a whole‐room metabolic chamber. NEAT was estimated by accelerometry and training reports, and EEE was calculated by subtraction. TEE and its components did not differ between amenorrhoeic and non‐amenorrhoeic runners. Weekly running distance did not correlate with TEE but was negatively linked to NEAT and positively to EEE, whereas its negative associations with RMR and SMR were attenuated after adjustment for body composition. Similar directional associations were observed in both groups, but the study was not powered to test effect modification by menstrual status. These findings are consistent with the constrained TEE model and suggest that higher training volume may be accompanied by a reallocation of energy expenditure among its components in chronically trained runners.

**Abbreviations**
ANCOVA: analysis of covariance
BM: body mass
DIT: diet‐induced thermogenesis
DLW: doubly labeled water
EE: energy expenditure
EEE: exercise energy expenditure
ELISA: enzyme‐linked immunosorbent assay
FDR: false discovery rate
FFM: fat‐free mass
FM: fat mass
FQ: food quotient
METs: metabolic equivalents
NEAT: non‐exercise activity thermogenesis
PAL: physical activity level (calculated as TEE ÷ RMR)
RMR: resting metabolic rate
SD: standard deviation
SMR: sleeping metabolic rate
TBW: total body water
TEE: total energy expenditure
VCO_2_
: carbon dioxide production
VO_2_
: oxygen consumption

## 1 Introduction

Competitive runners strategically regulate their body weight to optimize race performance by balancing energy intake and expenditure [1]. Total energy expenditure (TEE) primarily consists of resting metabolic rate (RMR), diet‐induced thermogenesis (DIT), non‐exercise activity thermogenesis (NEAT), and exercise energy expenditure (EEE). RMR, a major component of TEE, is largely determined by body mass (BM) and composition, whereas DIT depends on food quantity and composition and accounts for approximately 10% of TEE [2]. Thus, many studies have demonstrated that TEE increases additively with physical activity [3]. However, a unique study involving both men and women (16 each) reported that TEE did not increase additively with greater running distance. Instead, TEE appeared to be regulated through reductions in sleeping metabolic rate (SMR) [4]. This phenomenon is referred to as the constrained TEE model, in which increased EEE may be compensated for by reducing other components of TEE [5, 6]. Although conclusive evidence is lacking, the constrained TEE model may also apply to athletes, as chronic training under low energy intake, such as low energy availability, could induce similar adaptations. If TEE in athletes is constrained, components such as RMR may be suppressed to maintain energy balance [5].

RMR represents the energy required to sustain physiological functions, including reproduction and immunity, in humans. A few studies have reported that RMR in runners with amenorrhoea is lower than that in menstruating runners [7, 8], while another study indicated no difference in RMR according to menstrual status in the metabolic chamber [9, 10]. One potential explanation for these discrepancies is variation in the indirect calorimetry method [11, 12, 13, 14]. Whole‐room metabolic chambers are considered highly accurate tools for assessing energy metabolism, with low intra‐individual coefficients of variation for both RMR and SMR reported in previous whole‐room metabolic chamber‐based studies. The reported intra‐individual coefficients of variation were 3.7%–3.8% for RMR and 1.8%–3.3% for SMR [9, 15, 16, 17]. These advantages make metabolic chambers useful for detecting subtle differences and variations in RMR suppression associated with reproductive functions. Using this approach, SMR and RMR did not differ between amenorrhoeic and menstruating runners in the follicular phase [9], although chronic exercise training, such as that undertaken by athletes in daily life, may influence the temporal pattern of changes of EE during sleep. These findings suggest that amenorrhoea in adapted runners with chronic exercise training may not be explained solely by metabolic adaptation, as in the energy constraint model.

Measuring energy components under free‐living conditions is challenging because of the complexity and dynamics of physical activity and metabolism. Accelerometer‐based methods may not adequately capture physiological changes in energy metabolism, including energy adaptation, particularly during high‐intensity and complex activities [18]. The doubly labeled water (DLW) method is based on the turnover rates of two isotopes and provides a valuable approach for assessing TEE in free‐living athletes. Few studies have investigated the energy components of TEE using DLW that distinguish NEAT. A previous study demonstrated an interaction between sedentary energy expenditure (EE) and exercise energy compensation, assessed using the DLW method, in sedentary individuals who participated in a 24‐week aerobic exercise intervention; however, the constrained TEE model was not observed [19]. In contrast, Westerterp et al. demonstrated changes in energy components after approximately 10 months of continuous training [20, 21]; however, whether such adaptations persist in individuals engaged in chronic exercise training over several years or in those with amenorrhoea remains unclear. Therefore, it remains uncertain whether TEE and its components differ between amenorrhoeic and non‐amenorrhoeic athletes and whether the constrained TEE model occurs in athletes who maintain chronic training. Furthermore, the relationship between the components of TEE and physical activity, such as exercise training, running distance, and NEAT, under free‐living conditions has not been clarified, irrespective of menstrual status. Moreover, a low percentage of body fat has been identified as a contributing factor to reproductive dysfunction in women. However, runners with similar body fat percentages may exhibit varying reproductive functions, emphasizing the importance of investigating the energy components in these individuals.

Therefore, this study aimed to compare the components of TEE (SMR, RMR, NEAT, and EEE) between amenorrhoeic and non‐amenorrhoeic runners and examine the relationship between these energy components and physical activity, particularly weekly running distance.

## 2 Materials and Methods

Twenty volunteers (10 amenorrhoeic and 10 non‐amenorrhoeic runners; Table 1) were recruited for this study. Of these, 11 participants were also included in the previously reported cohort, comprising five amenorrhoeic and six non‐amenorrhoeic runners [9]. All volunteers were well‐trained runners with competitive experience in middle‐ or long‐distance (800–5000 m) running for at least 3 years (91.9 ± 26.4 months). These chronically trained runners were members of a university sports club and typically trained for more than 5 days/week. They had an athletic level above the Highly Trained/National Level, based on the athlete's participation classification framework. Participants with current medical conditions, metabolic diseases, or a history of smoking were excluded. The participants were not taking any medications affecting glucose or lipid metabolism, and none had thyroid or heart disease. Menstrual status was determined based on self‐reported history and personal interviews; no participant was diagnosed with gynecological disorders. Amenorrhoea was defined as three or fewer menstrual periods in the last 12 months or the absence of menstruation for more than 3 consecutive months. Participants who did not meet either criterion were classified as non‐amenorrhoeic runners (cycle length 25–38 days). None of the participants used hormonal contraceptives, including oral contraceptive pills, at the time of the study.

**TABLE 1:** Characteristics of study participants.

	Amenorrhoeic (*n* = 10)	Non‐amenorrhoeic (*n* = 10)	*p*
Age (years)	19.5 ± 1.4	19.2 ± 0.4	0.539
Height (cm)	157.4 ± 5.1	160.7 ± 4.2	0.130
Body mass (kg)	46.5 ± 5.2	48.4 ± 4.6	0.397
BMI (kg/m^2^)	18.8 ± 2.2	18.7 ± 1.5	0.905
%Body fat (%)	19.0 ± 5.1	21.3 ± 4.3	0.289
Fat mass (kg)	9.0 ± 3.4	10.4 ± 2.9	0.333
Fat‐free mass (kg)	37.5 ± 2.5	38.0 ± 2.5	0.661
Energy availability (kcal/kg/FFM/day)	32.4 ± 8.9	35.9 ± 12.0	0.473
PAL	2.20 ± 0.28	2.19 ± 0.25	0.928

*Note:* Data are expressed as mean ± SD. The characteristics of the participants were analyzed using Student's *t*‐test. There were no differences between amenorrhoeic and non‐amenorrhoeic runners.

Abbreviations: BMI, body mass index; FFM, fat‐free mass; PAL, physical activity level.

The experiment was performed for 8 days consecutively. The participants visited the laboratory on day 0 to determine the DLW dose and have the accelerometers fitted. Daily diet and physical activity were recorded for 7 days (days 1–7). On the final day, from the night of day 7 to the morning of day 8, the participants entered a metabolic chamber and remained there to measure their sleeping and resting metabolic rates. They were instructed to refrain from consuming caffeine‐containing beverages and alcohol for 3 days before entering the metabolic chamber. Participants did not undergo training to avoid influencing energy metabolism measurements and ate dinner 5 h before bedtime to minimize the effects of DIT on EE on day 7. The dinner consumed before the EE measurement consisted of typical Japanese food. The participants visited the laboratory on the evening of day 7 after dinner and entered the whole‐room indirect calorimeter after voiding urine. Participants were instructed to maintain a sitting posture until their habitual bedtime (23:00 or 00:00). They were awakened after 8 h by an intercom announcement and the lights being turned on. Resting metabolic rate was measured after the first morning urine sample was collected. Study protocols were explained to all participants, who provided written informed consent for all procedures. All procedures were conducted in accordance with the Declaration of Helsinki. This study was approved by the Institutional Review Board of the University of Tsukuba, Institutes of Health and Sports Sciences (Ref. Tai 021‐85).

This study was conducted without modifying the participants' daily lifestyle or training. Non‐amenorrhoeic participants were tested during the follicular phase. The participants notified the investigators when menstruation began, which was designated as day 1 of the menstrual cycle. TEE measurements of non‐amenorrhoeic participants were initiated between days 3 and 6 of the menstrual cycle to capture the follicular phase (2.8 ± 1.3 days) during a week under free‐living conditions. The TEE measurement period using DLW spanned the early follicular phase, whereas the metabolic chamber measurement, which focused on assessing SMR and RMR, was intended to be the late follicular phase.

### 2.1 Total Energy Expenditure and Body Composition Estimated by the Doubly Labeled Water Method

TEE and body composition were measured over 7 days using the DLW method. Participants were asked to maintain their initial BM and daily lifestyle. Participants ingested a weighed amount of ^18^O and ^2^H‐labeled water after providing a baseline urine sample (day 0). The dosing bottle was then rinsed twice with 40 mL of water. The dose volume was approximately 0.12 g/kg estimated total body water (TBW) for deuterium and approximately 1.8 g/kg estimated TBW for oxygen‐18 (99.8 atom% ^2^H_2_O and 10.0 atom% H_2_^18^O; Taiyo Nippon Sanso, Japan). Subsequent urine samples were collected at baseline before the DLW dose, after the DLW ingestion on the night of day 0, and in the morning and evening of days 1 and 7. All samples were stored frozen at −30° to −5°C in internally threaded polypropylene vials with screw cap wrapped in tight Parafilm M (Bemis Co. Inc., Oshkosh, WI, USA). An isotope ratio mass spectrometer (Sercon Isotope Ratio Mass Spectrometers, CF 20‐20; Sercon Ltd., Crewe, UK) was used to analyze isotope concentrations in the urine samples [22, 23]. TEE was calculated using the following equation: TEE (kcal/day) = *r*CO_2_ (L/day) × [1.106 + (3.94/FQ)] [23], where *r*CO2 represents carbon dioxide production, and FQ represents the food quotient. The food quotient was calculated as the percentage contribution of protein, fat, carbohydrates, and alcohol to total energy intake [24]. Well‐trained registered dietitians calculated energy and macronutrient intakes from dietary records and photographs using a computerized nutrient analysis software based on the 2016 version (7th rev.) of the Standard Tables of Food Composition in Japan (Excel Eiyou‐kun Version 8.0, Kenpakusha, Tokyo, Japan) [25]. DIT was estimated as 10% of TEE [2, 26]. PAL was calculated as DLW‐measured TEE divided by RMR [21, 27].

Height was measured early in the morning using a stadiometer. BM was measured using a digital scale on day 0 before the DLW dose and on day 8 during the morning fasting period after urine voiding. Fat‐free (FFM) mass and fat mass (FM) were calculated based on the 0.73 ratio of TBW to FFM [28].

### 2.2 Resting and Sleeping Metabolic Rate

The RMR and SMR were measured using a metabolic chamber (Fuji Medical Science Co. Ltd., Chiba, Japan) [9]. The airflow in the chamber was rigidly controlled and ventilated at rates of 80 or 110 L/min. The chamber temperature and relative humidity were set to 25°C and 50%–55%, respectively. Oxygen consumption (VO_2_) and carbon dioxide production (VCO_2_) in the outgoing air from the metabolic chamber were measured using an online process mass spectrometer (VG Prima δB; Thermo Electron, Winsford, UK) [9]. The EE and respiratory exchange ratio were calculated from the VO_2_, VCO_2_, and urinary nitrogen excretion measured using the Kjeldahl method, which was assumed to remain constant during calorimetry [29, 30, 31]. SMR was defined as the lowest mean EE observed over any consecutive 3‐h sleep interval, with the interval shifted in 30‐min increments, and was expressed as kcal/day. Subsequently, RMR was measured while participants rested supine on the bed for 30 min after a fasting period of at least 13.5 h. We excluded the first and last 5 min of the supine period to ensure that RMR was calculated during the stable supine time. Arousal energy expenditure was defined as the difference between RMR and SMR. Intra‐individual coefficient of variation and standard deviation (SD) for SMR and RMR, measured in 12 participants (nine males and three females, age: 21 ± 2 years, BMI: 20.4 ± 3.0 kg/m^2^) using a metabolic chamber, were 3.3% and 3.8%, and 61 and 72 kcal, respectively. The intra‐individual coefficients of variation and SD were similar to those reported previously [16, 17].

### 2.3 Daily Physical Activity Energy Expenditure and Exercise Energy Expenditure in Free‐Living Conditions

NEAT was estimated from metabolic equivalents (METs) measured with a triaxial accelerometer (Active style Pro HJA‐750C; OMRON Healthcare, Japan) worn on the waist, together with the participants' 7‐day training reports. For each epoch, activity energy expenditure above RMR was obtained by multiplying the measured RMR per minute by the METs value in excess of rest (METs − 1). The records were classified into training and non‐exercise periods using the training reports, and the non‐exercise activity energy expenditure above RMR was defined as NEAT; NEAT therefore included neither RMR nor DIT. DIT was estimated separately as 10% of TEE [2, 26]. EEE was calculated as EEE (kcal/d) = TEE – DIT − RMR − NEAT. Energy availability was calculated as (Energy intake − EEE)/FFM. Weekly running distance was assessed over 7 days according to each participant's individual training plan using a running wearable smartwatch (Garmin Forerunner; Garmin Japan Ltd., Japan).

### 2.4 Urinary Hormone Assays

Urinary 17β‐estradiol and progesterone concentrations were determined using the commercially available competitive enzyme immunoassay kits described below in accordance with the manufacturer's instructions, using pooled nocturnal urine collected throughout an 8‐h sleep period. An initial pilot assay was conducted using aliquots of the urine samples at the dilution factors recommended by the manufacturer. Because several samples yielded values outside the respective standard‐curve ranges, additional dilution factors were evaluated. Based on the pilot assay results, final dilution factors of 1:6 for the 17β‐estradiol assay and 1:60 for the progesterone assay were selected. In the final assays, all standards and urine samples were measured in duplicate, and the mean of the duplicate measurements was used for analysis. Concentrations were calculated using four‐parameter logistic standard curves and corrected for the respective dilution factors. All measured values fell within the respective standard‐curve ranges; therefore, no additional dilution, reassay, or extrapolation beyond the analytical ranges was required. Only the results obtained from the final assays were used for the statistical analysis. Total 8‐h urinary hormone excretion was calculated by multiplying the measured hormone concentration by the total urine volume collected and was expressed as ng/8 h. The correlation between the two hormones was examined using concentrations, as multiplying both by the same urine volume would inflate their association.

Urinary 17β‐estradiol was measured using a DetectX 17β‐Estradiol Enzyme Immunoassay Kit (catalogue no. K030‐H1; Arbor Assays, Ann Arbor, MI, USA). The manufacturer‐reported analytical sensitivity was 39.6 pg/mL, and the standard curve ranged from 39.06 to 10 000 pg/mL. The manufacturer‐reported intra‐ and inter‐assay coefficients of variation were 3.9%–7.3% and 3.6%–13.8%, respectively. Manufacturer validation using human urine demonstrated a mean recovery of 106.4% and good linearity (*R* ^2^ = 0.9995). Urinary progesterone was measured using a DetectX Progesterone ELISA Kit (catalogue no. K025‐H1; Arbor Assays, Ann Arbor, MI, USA). The manufacturer‐reported analytical sensitivity was 25.3 pg/mL, and the standard curve ranged from 50 to 3200 pg/mL. The manufacturer‐reported intra‐ and inter‐assay coefficients of variation were 3.5%–7.6% and 5.6%–6.7%, respectively. Manufacturer validation using human urine demonstrated a mean recovery of 95.3% and good linearity (*R* ^2^ = 0.9944).

### 2.5 Statistical Analysis

Data are presented as mean ± SD. All analyses were performed using IBM SPSS Statistics for Windows, version 28.0 (IBM Corp., Armonk, NY, USA). The statistical significance level was set at *p* < 0.05 for all analyses. The assumptions of normality and homogeneity of variance were confirmed for all variables. The relationship between EE and body composition was examined using Pearson's correlation. Partial correlations between running distance and each energy expenditure component (RMR, SMR, NEAT, and EEE) were additionally computed with adjustment for FFM and FM using the ppcor package in R. As a sensitivity analysis, the partial correlations were repeated on natural log‐transformed variables. To account for multiple testing in the correlation analyses, false discovery rate (FDR) correction was applied using the Benjamini–Hochberg procedure. The correction was applied to the family of five correlations between weekly running distance and TEE, RMR, SMR, NEAT, and EEE. The partial correlations presented as sensitivity analyses were not included in this correction.

Multiple linear regression analyses were conducted using the forced‐entry method to examine the independent associations of FFM and weekly running distance with RMR, SMR, and TEE. These predictors were selected a priori based on this study's hypothesis. The number of predictors was limited given the sample size (*n* = 20). Menstrual status was not included as a predictor or effect modifier because between‐group comparisons addressed the first study aim, whereas the regression analyses examined associations in the full cohort. Given the limited sample size, the models were restricted to the two prespecified predictors. For each outcome, the model was specified as follows: outcome = *β*_0_ + *β*_1_(FFM) + *β*_2_(weekly running distance) + *ε*. Both predictors were entered simultaneously, with no hierarchical ordering. Predictors were not mean‐centred because the models included no interaction terms. Linearity and homoscedasticity were assessed visually using plots of standardized residuals against standardized predicted values, while residual normality was assessed using histograms and normal P–P plots of the standardized residuals. Thus, model assumptions were evaluated graphically. Multicollinearity was assessed using variance‐inflation factors. Potentially influential observations were evaluated using studentized deleted residuals, Cook's distance, leverage values, and DFBETAs.

Differences in the absolute values of TEE, RMR, SMR, arousal EE, NEAT, and EEE between groups were analyzed using an analysis of covariance (ANCOVA), with FFM and FM as covariates, whereas Student's *t*‐tests were used to examine differences in the relative contributions of each energy expenditure to TEE between amenorrhoeic and non‐amenorrhoeic runners.

## 3 Results

The participants had similar physical characteristics (Table 1). The initial BM (47.5 ± 4.9 kg) and final BM (47.3 ± 4.9 kg) values were similar. Significant differences in the urinary excretion of 17β‐estradiol and progesterone during sleep were observed between groups (17β‐estradiol: *p* = 0.010; progesterone: *p* = 0.001; Table 2). A significant correlation was found between the urinary excretion of 17β‐estradiol and progesterone (pg/mL; slope, 0.0642; intercept, 25.583; *r* = 0.815; *p* < 0.001). There was no difference in age at menarche between amenorrhoeic (14.7 ± 1.9 years) and non‐amenorrhoeic runners (13.6 ± 2.2 years). Additionally, seven of the ten amenorrhoeic runners had irregular menstruation or amenorrhoea lasting more than 2 years (average duration 29.1 ± 18.4 months; range 5–57 months).

**TABLE 2:** Urinary 17β‐estradiol and progesterone excretion during the sleep period.

	Amenorrhoeic (*n* = 10)	Non‐amenorrhoeic (*n* = 10)	*p*
17β‐Estradiol (ng/8 h)	543 ± 235	1012 ± 462	0.010
Progesterone (ng/8 h)	9240 ± 4154	15 422 ± 2786	0.001

*Note:* Data are expressed as mean ± SD. Urinary 17β‐estradiol and progesterone are expressed as total urinary excretion during the 8‐h sleep period (ng/8 h). Between‐group differences in urinary hormone excretion were analyzed using Student's *t*‐test.

**FIGURE 1:**
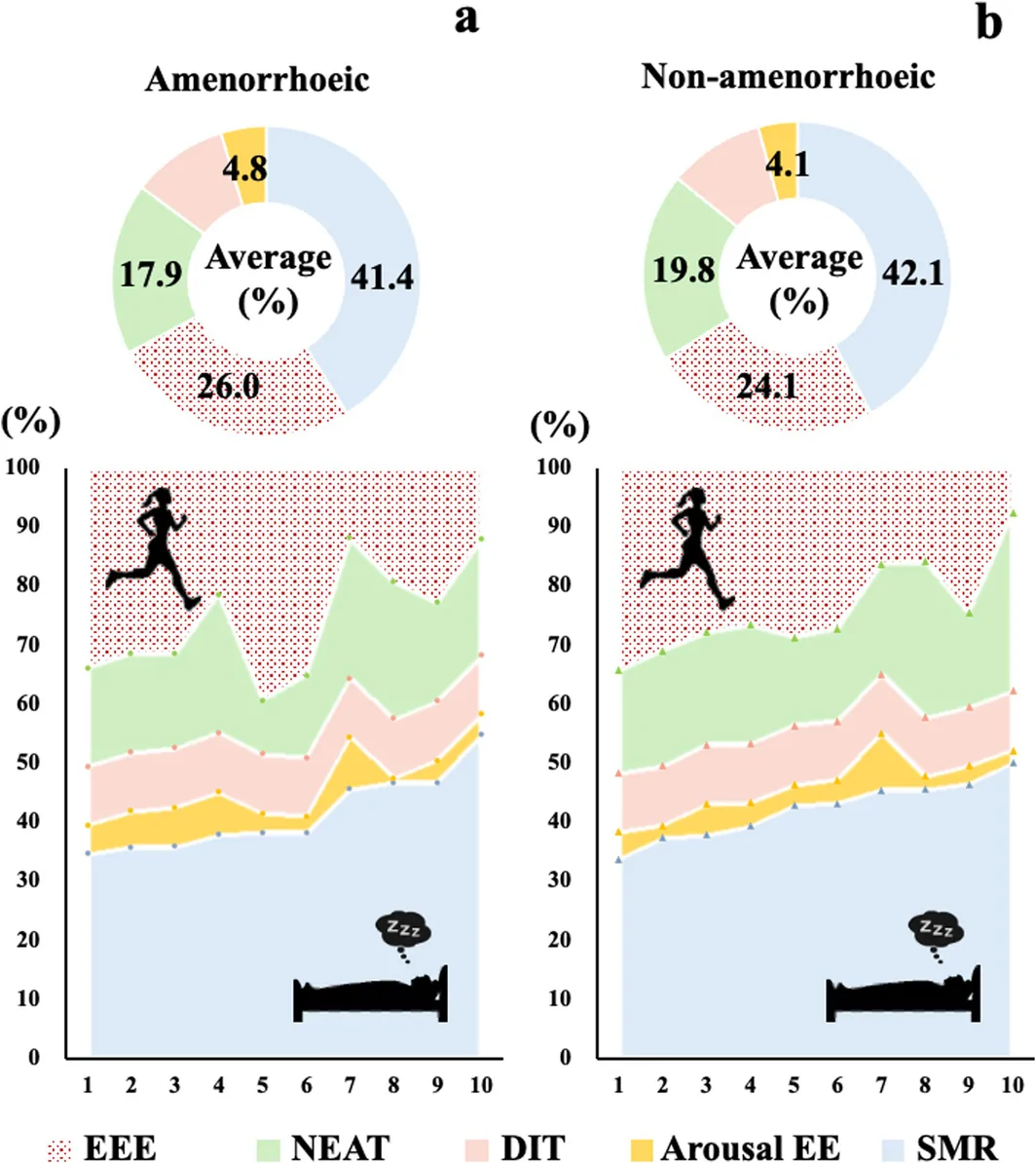
Components of total energy expenditure in female runners. Sunburst charts show the average percentage of each energy component in the TEE for (a) amenorrhoeic and (b) non‐amenorrhoeic runners. The area charts show the percentage of each energy component relative to the TEE in the individual data. Abbreviations: arousal EE, arousal energy expenditure calculated by the difference between resting and sleeping metabolic rates; DIT, diet‐induced thermogenesis; EEE, exercise energy expenditure estimated by a combination of doubly labeled water, metabolic chamber, and accelerometer; NEAT, non‐exercise activity thermogenesis, estimated from accelerometer‐based metabolic equivalents above RMR during non‐exercise periods; SMR, sleeping metabolic rate measured with a metabolic chamber; TEE, total energy expenditure obtained by doubly labeled water.

No significant differences were observed in TEE, RMR, SMR, arousal EE, NEAT, or EEE (Table 3). Each component of TEE also showed no significant differences between amenorrhoeic (RMR, 46.1% ± 6.3%; SMR, 41.4% ± 6.6%; arousal EE, 4.8% ± 2.4%; NEAT, 17.9% ± 4.7%; EEE, 26.0% ± 9.7%) and non‐amenorrhoeic runners (RMR, 46.2% ± 5.3%; SMR, 42.1% ± 5.0%; arousal EE, 4.1% ± 2.2%; NEAT, 19.8% ± 4.9%; EEE, 24.1% ± 8.2%) (Figure 1a,b).

**TABLE 3:** Comparison of unadjusted and adjusted energy expenditure components between groups.

Outcome	Unadjusted (raw)		Adjusted (ANCOVA)				
	Amenorrhoeic (*n* = 10)	Non‐amenorrhoeic (*n* = 10)	Amenorrhoeic EMM	Non‐amenorrhoeic EMM	Between‐group difference (95% CI)	*p*	Partial *η* ^2^
TEE (kcal/d)	2506 ± 276	2538 ± 258	2509 ± 79	2535 ± 79	−26.5 (−267, 215)	0.819	0.003
RMR (kcal/d)	1145 ± 119	1161 ± 67	1161 ± 22	1145 ± 22	15.3 (−52, 82)	0.635	0.014
SMR (kcal/d)	1029 ± 159	1059 ± 75	1048 ± 31	1041 ± 31	6.9 (−87, 101)	0.879	0.002
Arousal EE (kcal/d)	115 ± 52	102 ± 49	113 ± 16	104 ± 16	8.3 (−40, 57)	0.721	0.008
NEAT (kcal/d)	444 ± 111	499 ± 111	447 ± 37	496 ± 37	−48.8 (−161, 63)	0.370	0.050
EEE (kcal/d)	666 ± 283	625 ± 248	650 ± 81	641 ± 81	9.4 (−237, 256)	0.937	< 0.001

*Note:* Data are expressed as mean ± SD for unadjusted values and as estimated marginal means ± SE for adjusted values. Between‐group differences were assessed using ANCOVA with fat‐free mass and fat mass as covariates, with adjusted differences calculated as amenorrhoeic minus non‐amenorrhoeic.

Abbreviations: arousal EE, arousal energy expenditure estimated by the difference between RMR and SMR; CI, confidence interval for the adjusted between‐group difference; EEE, exercise energy expenditure estimated using doubly labeled water, metabolic chamber and accelerometer; EMM, estimated marginal mean; NEAT, non‐exercise activity thermogenesis, estimated from accelerometer‐based metabolic equivalents above RMR during non‐exercise periods; RMR, resting metabolic rate measured with metabolic chamber; SE, standard error; SMR, sleeping metabolic rate measured with metabolic chamber; TEE, total energy expenditure obtained by doubly labeled water.

A strong correlation was observed between SMR and RMR (slope, 1.197; intercept, 336.3; *r* = 0.927; *p* < 0.001). A significant correlation was found between RMR and BM (slope, 12.98; intercept, 537.5; *r* = 0.670; *p* = 0.001), RMR and FM (slope, 22.25; intercept, 936.6; *r* = 0.739; *p* < 0.001), SMR and BM (slope, 16.57; intercept, 258.8; *r* = 0.661; *p* = 0.002), and SMR and FM (slope, 26.47; intercept, 787.0; *r* = 0.680; *p* = 0.001). RMR and SMR did not significantly correlate with the FFM (RMR, *p* = 0.102; SMR, *p* = 0.055). Running distance was not significantly correlated with FM (*r* = −0.416, *p* = 0.068) or FFM (*r* = −0.084, *p* = 0.724).

**FIGURE 2:**
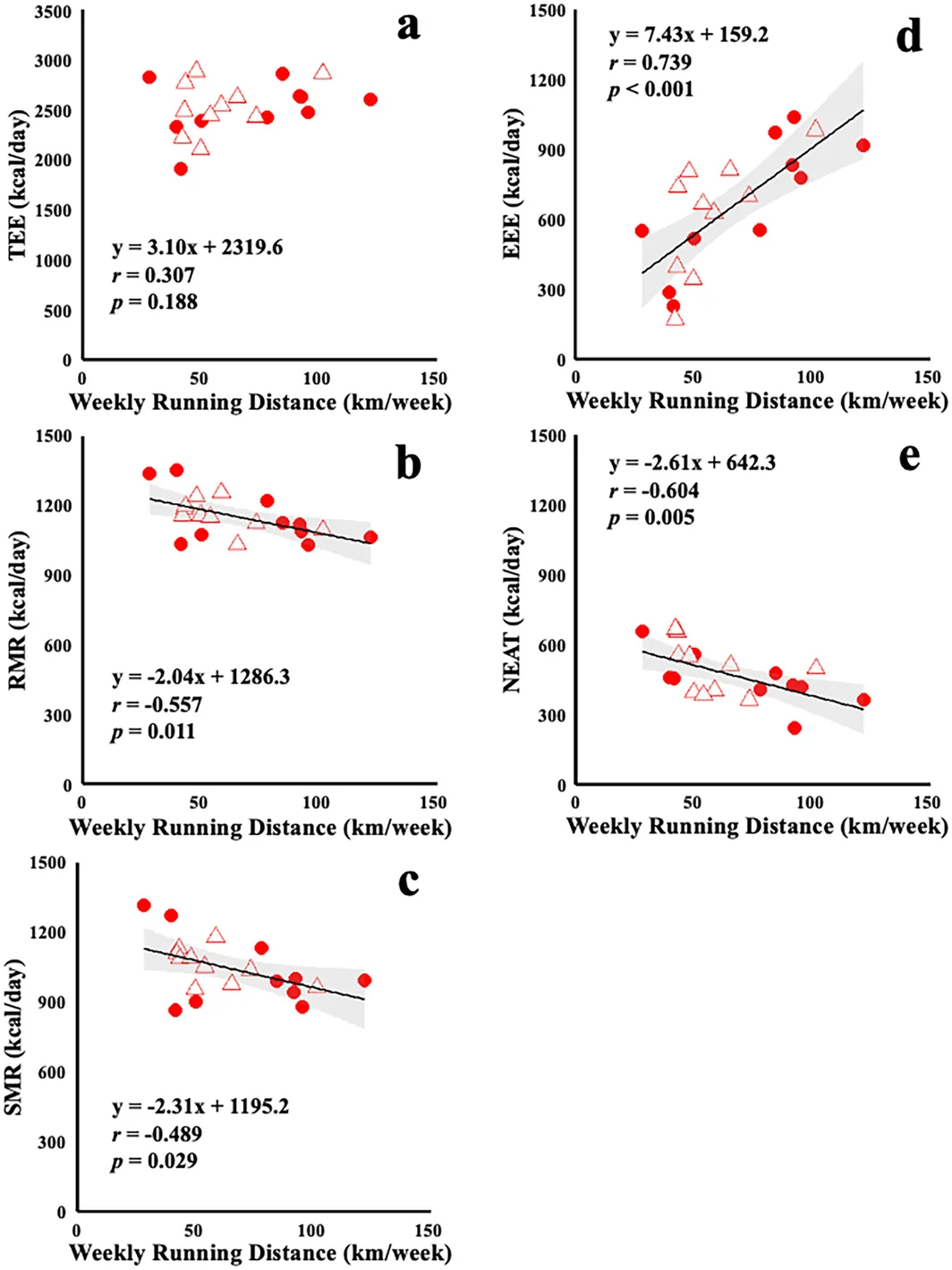
Relationship between running distance and components of total energy expenditure among all female runners. Scatter plots represent the relationship between weekly running distance and (a) TEE, (b) RMR, (c) SMR, (d) EEE, and (e) NEAT. The red circles indicate amenorrhoeic runners, and the white triangles with a red border indicate non‐amenorrhoeic runners. Symbols indicate menstrual status (amenorrhoeic, *n* = 10; non‐amenorrhoeic, *n* = 10), whereas all correlations and fitted regression lines are based on the pooled sample (*n* = 20). Gray areas represent 95% confidence intervals. Abbreviations: EEE, exercise energy expenditure; NEAT, non‐exercise activity thermogenesis; RMR, resting metabolic rate; SMR, sleeping metabolic rate; TEE, total energy expenditure.

PAL was similar in amenorrhoeic (2.20 ± 0.28, range 1.72–2.54) and non‐amenorrhoeic runners (2.19 ± 0.25, range 1.82–2.61). Weekly running distance was 72.7 ± 30.7 km (range 28.2–122.4 km) for amenorrhoeic runners and 58.1 ± 18.6 km (range 42.2–102.0 km) for non‐amenorrhoeic runners, with no significant between‐group difference (*p* = 0.217). TEE did not correlate with weekly running distance (*p* = 0.188; Figure 2a). In contrast, RMR and SMR were significantly negatively correlated with weekly running distance (RMR: slope, −2.04; intercept, 1286.3; *r* = −0.557; *p* = 0.011; Figure 2b; SMR: slope, −2.31; intercept, 1195.2; *r* = −0.489; *p* = 0.029; Figure 2c). After adjustment for FFM and FM, these associations were attenuated and no longer significant (RMR: partial *r* = −0.414, *p* = 0.088; SMR: partial *r* = −0.340, *p* = 0.167; Figure S2a). EEE was positively correlated with weekly running distance (slope, 7.43; intercept, 159.2; *r* = 0.739; *p* < 0.001; Figure 2d), whereas NEAT was negatively correlated with weekly running distance (slope, −2.61; intercept, 642.3; *r* = −0.604; *p* = 0.005; Figure 2e). These associations remained essentially unchanged after adjustment for FFM and FM (EEE: partial *r* = 0.718, *p* < 0.001; NEAT: partial *r* = −0.639, *p* = 0.004; Figure S2a). Results were similar when analyses were repeated on log‐transformed variables; the associations of running distance with NEAT and EEE remained significant, whereas those with RMR and SMR were sensitive to the functional form (RMR: partial *r* = −0.493, *p* = 0.037; SMR: partial *r* = −0.417, *p* = 0.085 on the log scale). The FDR–adjusted *p*‐values and the correlations between NEAT and the other energy expenditure components are presented in Tables S2 and S3, respectively.

Multiple regression analyses were performed to identify significant predictors of RMR, SMR, and TEE (Table 4). Weekly running distance was selected as a significant predictor of both RMR and SMR. FFM showed a trend for either outcome (RMR: *p* = 0.092; SMR: *p* = 0.051). In contrast, for TEE, FFM was a significant predictor (*p* = 0.026), whereas weekly running distance showed a trend (*p* = 0.099).

**TABLE 4:** Forced‐entry multiple regression of energy expenditure among all female runners.

Outcome	Predictor	Unstandardised coefficients		Standardized coefficient	*t‐*value	Coefficient *p‐*value	95% CI for *B*		VIF	*R* ^2^	Adjusted *R* ^2^	*F*‐value	Model *p‐*value	RSE
		*B*	SE	*β*			Lower	Upper						
RMR	Constant	797.984	277.160	—	2.879	0.010	213.228	1382.739						
	Weekly running distance	−1.936	0.678	−0.529	−2.855	0.011	−3.367	−0.505	1.007					
	FFM	12.774	7.144	0.332	1.788	0.092	−2.299	27.847	1.007					
	Model									0.420	0.351	6.148	0.010	76.042
SMR	Constant	438.831	365.600	—	1.200	0.246	−332.517	1210.179						
	Weekly running distance	−2.151	0.895	−0.455	−2.405	0.028	−4.039	−0.264	1.007					
	FFM	19.789	9.424	0.397	2.100	0.051	−0.094	39.672	1.007					
	Model									0.396	0.324	5.561	0.014	100.307
TEE	Constant	344.752	821.555	—	0.420	0.680	−1388.578	2078.081						
	Weekly running distance	3.509	2.010	0.348	1.746	0.099	−0.732	7.750	1.007					
	FFM	51.665	21.177	0.486	2.440	0.026	6.985	96.345	1.007					
	Model									0.329	0.250	4.170	0.034	225.403

*Note:* Data are presented as regression coefficients with standard errors. Multiple regression analyses were performed to identify significant predictors of RMR, SMR, and TEE. Fat‐free mass and weekly running distance were entered simultaneously using the enter method. For all models, the residual degrees of freedom were 17; the overall *F* tests had 2 numerator degrees of freedom. RSE is expressed in kcal/day. B for weekly running distance represents the estimated difference in the outcome, expressed in kcal/day, per additional 1 km/week of running distance, after adjustment for fat‐free mass. B for fat‐free mass represents the estimated difference in the outcome per additional 1 kg of fat‐free mass, after adjustment for weekly running distance.

Abbreviations: *β*, standardized partial regression coefficient; *B*, partial regression coefficient; CI, confidence interval for *B*; FFM, fat‐free mass estimated using the doubly labeled water method; RMR, resting metabolic rate; RSE, residual standard error; SE, standard error; SMR, sleeping metabolic rate; TEE, total energy expenditure obtained using doubly labeled water; VIF, variance‐inflation factor.

## 4 Discussion

This study evaluated the components of TEE in female runners and examined their relationships with menstrual status and physical activity under free‐living conditions. Our primary findings indicated that TEE and its components, RMR, SMR, NEAT, and EEE, did not differ between amenorrhoeic and non‐amenorrhoeic runners. However, weekly running distance was negatively correlated with NEAT and positively with EEE, and negatively correlated with RMR and SMR, with similar directional associations observed in both menstrual status groups (Figure 2). These data suggest that elevated training volume may be accompanied by a reallocation of activity‐related energy expenditure (lower NEAT and higher EEE) in these runners (Figure 1). Although the negative associations between weekly running distance and RMR/SMR did not reach statistical significance when analyzed within the smaller subgroups (*n* = 10 each), the direction and magnitude of the correlation coefficients remained consistent across both groups (Figure S1a). Therefore, our findings based on the pooled samples suggest that the energy expenditure components, particularly NEAT and EEE, were associated with training volume, while the extent to which these associations differ by menstrual status remains uncertain, as menstrual status was not included as an effect modifier and the study was not powered to test effect modification. The negative associations with RMR and SMR, although attenuated after adjustment for body composition, were in the same direction, suggesting a tendency toward lower resting metabolism with higher training volume.

Despite comparable energy expenditure profiles (Figure 1), weekly running distance was negatively associated with NEAT and positively with EEE, and these associations were independent of body composition (Figure S2a). Running distance was also negatively associated with RMR and SMR (Figure 2b,c), although these associations were attenuated after adjustment for FFM and FM, as running distance tended to be associated with FM but not FFM. These findings suggest that chronic endurance training may induce a reallocation of energy expenditure to maintain energy balance under conditions of elevated energy flux, even in the absence of overt reproductive dysfunction. These findings align with the constrained TEE model proposed by Pontzer, which suggests that TEE plateaus despite increased physical activity in hunter‐gatherer populations because of compensatory reductions in other energy components [5, 6]. However, recent evidence on this model remains mixed; while Howard et al. [32] reported a positive linear relationship between physical activity and TEE across a wide range of levels, supporting an additive rather than constrained model, our results in highly trained runners show higher EEE and lower NEAT with higher training volume, indicating a trade‐off between exercise and non‐exercise activity. The foundational work by Westerterp et al. further refines this view, showing that reductions in SMR are closely linked to energy balance and BM change, and that energy efficiency can increase over time with training, suggesting adaptive improvement in exercise economy rather than a linear increase in energy expenditure [20, 21]. The reallocation of activity‐related energy expenditure, together with a tendency toward lower RMR and SMR, may indicate an energy saving response to maintain daily energy needs within a limited maximum. This is consistent with the duration‐dependent metabolic ceiling of ~2.5 × BMR observed in ultra‐endurance athletes over long periods [33]. In our female runners, PAL (calculated as TEE/RMR) approaches this sustainable limit noted by Best et al. [33] (amenorrhoea 2.20 and non‐amenorrhoeic 2.19). Within this constrained range, greater training volume, indexed by weekly running distance, was associated with lower NEAT and a tendency toward lower RMR and SMR.

Importantly, while previous studies have demonstrated a reduction in RMR in amenorrhoeic athletes [7], our findings suggest that these reductions may not be restricted to athletes with amenorrhoea. Instead, the tendency toward lower RMR and SMR, together with the reallocation of NEAT and EEE, may represent a general adaptation to high training volumes in these runners. This raises the possibility that other physiological systems, such as the immune system or thyroid function, could be affected by chronic energy constraints. The findings of the present study are consistent with those of Westerterp et al. [20, 21], who showed that even when physical activity increases, the body may compensate for elevated energy expenditure by reducing other components of energy expenditure. While Howard et al. [32] found no significant association between physical activity and biomarkers of immune or reproductive function, a broader meta‐analysis by Pontzer and Trexler [34] reported that reductions in BMR and SMR contribute to energy compensation, particularly in aerobic interventions and longer‐duration studies. Although our study did not directly measure immune markers, Klasson et al. [35] reported cross‐sectional associations between physical activity and thyroid hormones, inflammatory markers, and immune‐related biomarkers. However, these cross‐sectional findings do not provide direct evidence that energy is reallocated away from immune or thyroid function under constrained energy conditions. Therefore, the potential involvement of these physiological systems, such as the immune system or thyroid function, in chronic energy constraints remains speculative; these systems were not examined in the present study and warrant further investigation.

Another key implication of our findings is the need to account for recent training or chronic training habits when interpreting RMR. Variability in the prior‐week training volume may contribute to the inconsistent findings across studies comparing RMR between athletes with and without menstrual disturbances. Our results suggest that, beyond short‐term pre‐measurement fasting or rest periods, several days of recorded activity are required to properly interpret energy expenditure in endurance‐trained populations. Furthermore, energy availability, central to the female athlete triad frameworks [36], is inherently difficult to quantify in free‐living individuals [37, 38] because the EEE and energy intake vary substantially by activity type and measurement approach [18, 37]. Because well‐trained runners usually have high energy efficiency during exercise, energy availability (kcal/kg FFM/day) could be underestimated when EEE is not derived from objective physiological measurements, such as TEE assessed using the DLW method. Thus, expressing energy intake or expenditure per kilogram of FFM may be inappropriate, and expressing it as ‘kcal/kg FFM/day’ is not a meaningful parameter for evaluating the energy saving of the TEE model. In light of these conceptual and measurement challenges, our study evaluated a constrained TEE model in free‐living conditions using both a metabolic chamber and DLW. Based on these measurements, the female runners in this study, who had experience with a long training period (mean training duration 92 ± 26 months), may have had higher training‐related energy efficiency. Notably, the PAL of our runners (≈2.2) was considerably higher than that of the exercise‐intervention cohorts summarized by Pontzer and Trexler (1.58–1.67) [34] and approached the proposed sustainable ceiling of ~2.5, indicating that our participants operated at a high energy flux at which compensation is more likely to be evident. Pontzer and Trexler [34] suggested that such compensation may take approximately 15 weeks or more to be observed, which aligns with the chronic nature of our participants' training. Consistent with our findings, Westerterp et al. [20] reported that average daily metabolic rate remained relatively stable despite a substantial increase in endurance‐training volume, while SMR decreased at the highest training load, suggesting that increases in exercise‐related expenditure may be partly offset by reductions in other components of daily energy expenditure. In our regression model, SMR and RMR declined by approximately 2 kcal for every 1 km increase in weekly running distance in models including FFM as a predictor (Table 4). Our group previously reported no significant differences related to the absence of menstruation [9]. Extending these reports, our findings suggest that the TEE components, particularly NEAT and EEE, are associated with running distance, whereas differences by menstrual status were not detected.

The relationship between energy expenditure and FFM generally has a positive intercept; consequently, this positive intercept results in higher values for participants with lower FFM than expected. In our female runners, FFM was not a strong predictor of RMR and SMR. Both groups had nearly identical FFM, and the narrow FFM range may therefore have attenuated associations between RMR or SMR and FFM. Moreover, although FM did not significantly differ between menstrual status groups, we observed a positive correlation between FM and 17β‐estradiol excretion (*r* = 0.553; *p* = 0.012), echoing earlier findings that low FM is associated with reproductive suppression [39]. However, the variation in reproductive function among athletes with similar FM highlights the importance of directly evaluating energy balance and TEE components, rather than relying solely on body composition as determinants of menstrual function.

The present study had some limitations. First, the cross‐sectional design precludes causal inference regarding the relationship between training load and components of TEE. Moreover, cross‐sectional correlations cannot formally distinguish a constrained model from an additive one, because both predict that energy expenditure components covary with physical activity; a formal test requires quantifying energy compensation against the expected change in expenditure in longitudinal or interventional designs [34]. Our findings should therefore be regarded as consistent with, rather than confirmatory of, a constrained model. Second, we acknowledge the limited sample size, which led to a loss of statistical significance in the subgroup correlation analyses (Figure S1a). In addition, the negative associations of weekly running distance with RMR and SMR were sensitive to the functional form (significant on the log scale but not on the linear scale), whereas the associations with NEAT and EEE were robust; this further indicates that the RMR and SMR associations are weak and dependent on model specification. However, effect sizes across all TEE components were consistently small and smaller than the detectable differences estimated by sensitivity analysis, suggesting that clinically meaningful physiological differences between groups were unlikely (Tables S1 and S2). In the future, a large database, such as the IAEA database, may provide further insight into these associations [40]. Third, prolonged 24‐h confinement in the metabolic chamber was not feasible because of the participants' demanding academic and training schedules as collegiate student athletes. Consequently, the chamber was used strictly for a 10‐h overnight period to precisely capture SMR and RMR. The lack of 24‐h chamber data presented two methodological constraints: (i) we could not directly compare chamber‐derived TEE with DLW‐derived TEE, and (ii) fine estimations of DIT were not feasible. Although participants consumed dinner 5 h prior to bedtime to minimize DIT effects during the chamber stay, we relied on a 10% assumption, whereas the actual DIT might vary in athletes consuming higher amounts of protein. Finally, because of these methodological constraints and the specific focus on highly trained female runners, the findings should be interpreted with caution when generalizing them to other athletic populations or training contexts.

This study demonstrated that neither TEE nor its components (RMR, SMR, NEAT, and EEE) differed significantly between runners with and without amenorrhoea. However, weekly running distance was negatively associated with NEAT and positively with EEE independent of body composition, whereas its negative associations with RMR and SMR were attenuated after adjustment for body composition. These findings are consistent with a constrained TEE model, in which a reallocation of energy expenditure components accompanies higher training volume, with similar patterns observed in both groups. Because cross‐sectional correlations cannot distinguish constrained from additive models, these associations should be interpreted as consistent with, rather than confirmatory of, energy compensation.

## 5 Perspective

This study suggests that components of TEE are associated with training volume in female runners. Although TEE and its components did not differ by menstrual status, greater weekly running distance was associated with lower NEAT and higher EEE, and with a tendency toward lower RMR and SMR, suggesting compensatory energy conservation. These findings are consistent with the constrained TEE model and suggest that chronic endurance training may be accompanied by a reallocation of components of TEE to maintain energy balance. Importantly, these associations showed similar directional patterns in both groups, although the study was not powered to test effect modification. Future longitudinal studies that quantify energy compensation against the expected change in expenditure are needed to determine whether these patterns represent beneficial efficiency or early signs of energetic strain in athletes.

## Author Contributions

A.U. and H.S. conceived and designed the study; A.U., E.K., Y.E., K.W., and H.S. conducted the experiments; A.U., K.Y., S.T., K.W., A.I., Y.H., and H.S. analyzed the data; A.U., E.K., Y.E., and H.S. interpreted the experimental results; A.U. and H.S. prepared the illustrations; A.U. and H.S. drafted the manuscript; All authors commented, edited, and revised the manuscript. All authors have read and approved the final version of this manuscript.

## Funding

This work was supported by the University of Tsukuba Basic Research Support Program Type S to H.S.; the JSPS KAKEN to A.U. (22J11154, 25KJ0427), H.S. (23H03279, 23KK0177, 26K02782).

## Ethics Statement

Study protocols were explained to all participants, who provided written informed consent for all procedures. All procedures were conducted in accordance with the Declaration of Helsinki. This study was approved by the Institutional Review Board of the University of Tsukuba, Institutes of Health and Sports Sciences (Ref. Tai 021‐85, 12/20/2024).

## Conflicts of Interest

The authors declare no conflicts of interest.

## Supporting information


**Figure S1a:** The association between weekly running distance and EEE, NEAT, RMR, SMR, or TEE within the amenorrhoeic group.
**Figure S1b:** The association between weekly running distance and EEE, NEAT, RMR, SMR, or TEE within the non amenorrhoeic group.
**Figure S2a:** Partial correlations between weekly running distance and energy expenditure components after adjustment for FFM and FM among all female runners (*n* = 20).
**Figure S2b:** Partial correlations between weekly running distance and energy expenditure components after adjustment for FM among all female runners (*n* = 20).
**Figure S2c:** Partial correlations between weekly running distance and energy expenditure components after adjustment for body mass among all female runners (*n* = 20).

**Table S1:** Sensitivity analysis and post hoc sample size estimates for between‐group comparisons of TEE components.
**Table S2:** Raw and FDR‐adjusted *p*‐values for correlation analyses between running distance and components of total energy expenditure.
**Table S3:** Pearson correlations between NEAT and TEE components and weekly running distance.

## Data Availability

Data supporting the findings of this study are available upon request from the corresponding author. The data are not publicly available because of privacy and ethical restrictions.
