# Supplementary material for: Running Distance Is Associated With Components of Total Energy Expenditure in Amenorrhoeic and Non‐Amenorrhoeic Endurance Runners
Source: Scand J Med Sci Sports. 2026 Aug 31;36(9):e70362. doi: 10.1111/sms.70362 (PMC13527886; doi:10.1111/sms.70362)
Supplement: Supplementary file 1 [file SMS-36-e70362-s001.pdf]

Supplementary Tables for

Running distance is associated with components of total energy expenditure in amenorrhoeic and non-amenorrhoeic endurance runners

Akiko Uchizawa et al.

Table S1. Sensitivity analysis and post hoc sample size estimates for between-group comparisons of TEE components

	Observed difference (kcal/day)	Pooled SD	Observed Cohen's d	Detectable difference (kcal/day)	Detectable Cohen's d	Required sample size per group (80% power)
**RMR**	16	97	0.166	128	1.325	545
**SMR**	30	124	0.241	165	1.325	274
**NEAT**	3	45	0.066	60	1.325	3605
**EEE**	41	266	0.154	353	1.325	663
**TEE**	32	267	0.120	354	1.325	1096

RMR, resting metabolic rate; SMR, sleeping metabolic rate; NEAT, non-exercise activity thermogenesis; EEE, exercise energy expenditure; TEE, total energy expenditure; SD, standard deviation.

Table S2. Raw and FDR-adjusted p-values for correlation analyses between running distance and components of total energy expenditure

	p-value	FDR-adjusted p-value
**EEE**	< 0.001	< 0.001
**NEAT**	0.005	0.012
**RMR**	0.011	0.018
**SMR**	0.029	0.036
**TEE**	0.188	0.188

FDR, false discovery rate (Benjamini-Hochberg procedure); EEE, exercise energy expenditure; RMR, resting metabolic rate; NEAT, non-exercise activity thermogenesis; SMR, sleeping metabolic rate; TEE, total energy expenditure obtained using doubly labelled water.

Table S3. Pearson correlations between NEAT and TEE components and weekly running distance

	RMR	SMR	EEE	TEE	Weekly running distance
**Pearson r**	0.317	0.351	−0.420	0.138	−0.604
**P-value**	0.173	0.129	0.065	0.563	0.005

RMR, resting metabolic rate; SMR, sleeping metabolic rate; NEAT, non-exercise activity thermogenesis; EEE, exercise energy expenditure; TEE, total energy expenditure.
