# Supplementary material for: Running Distance Is Associated With Components of Total Energy Expenditure in Amenorrhoeic and Non‐Amenorrhoeic Endurance Runners
Source: Scand J Med Sci Sports. 2026 Aug 31;36(9):e70362. doi: 10.1111/sms.70362 (PMC13527886; doi:10.1111/sms.70362)
Supplement: Supplementary file 2 [file SMS-36-e70362-s002.pdf]

Supplementary Figures for

Running distance is associated with components of total energy expenditure in amenorrhoeic and non-amenorrhoeic endurance runners

Akiko Uchizawa et al.

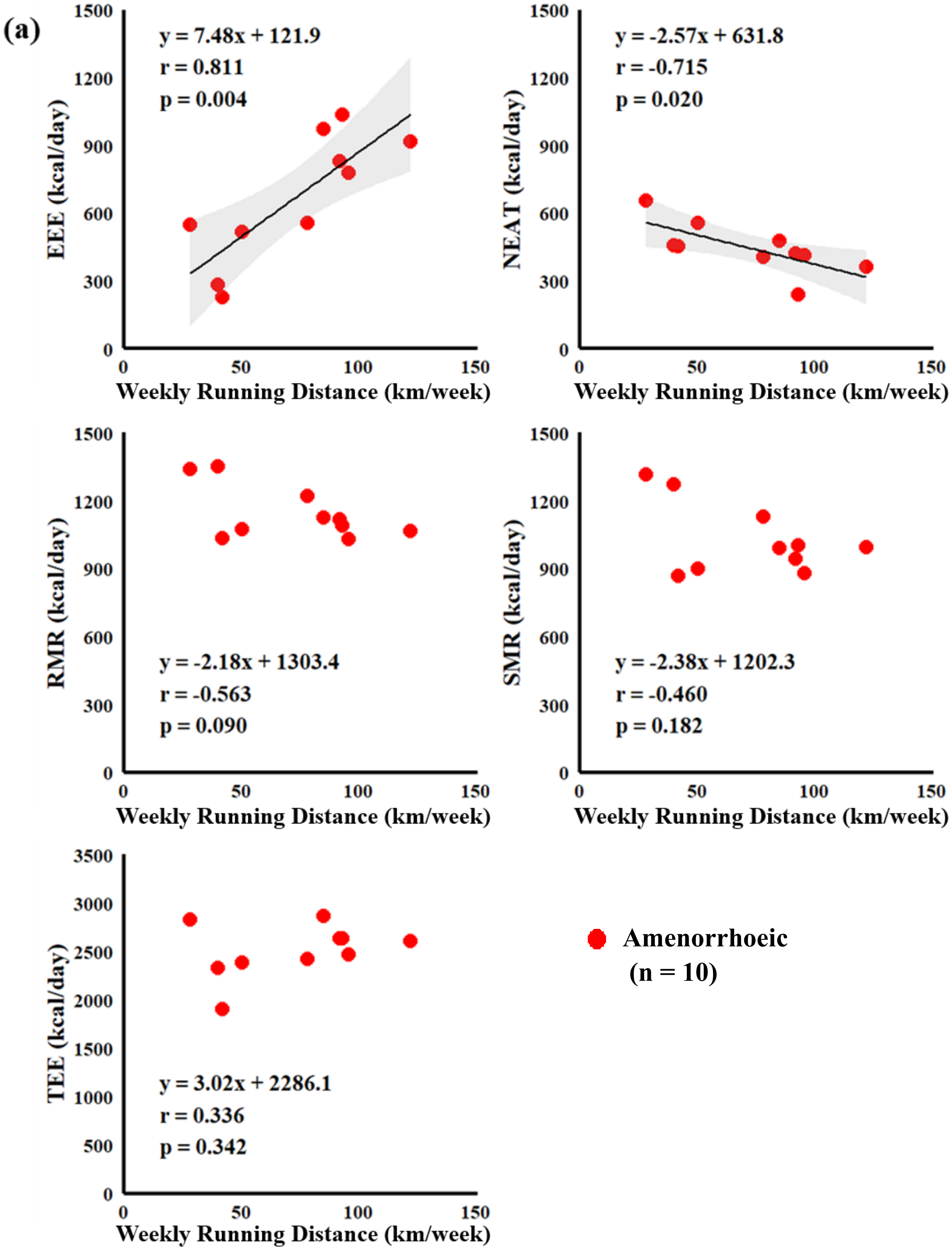

Figure S1. (a) The association between weekly running distance and EEE, NEAT, RMR, SMR, or TEE within the amenorrhoeic group.

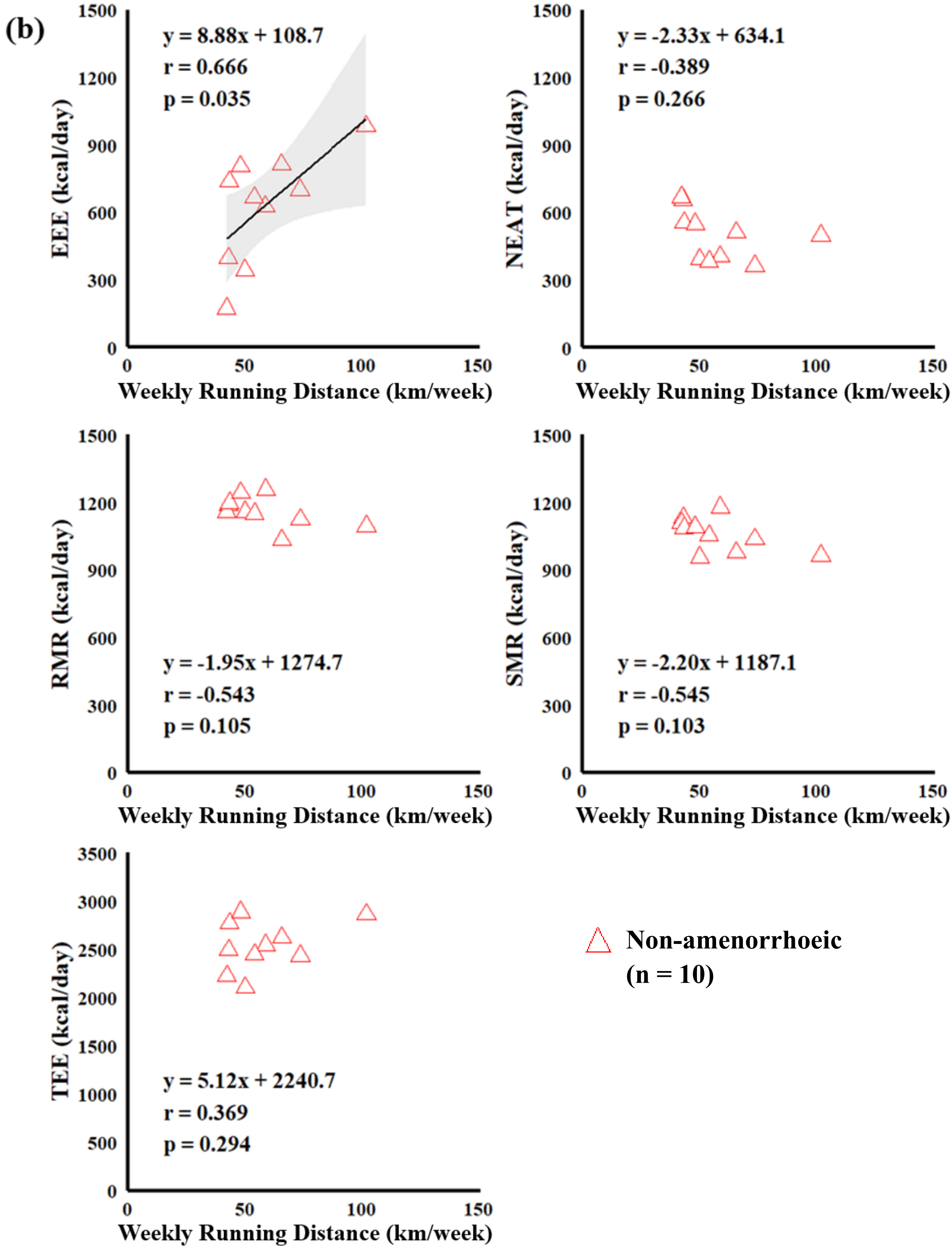

Figure S1. (b) The association between weekly running distance and EEE, NEAT, RMR, SMR, or TEE within the non-amenorrhoeic group.

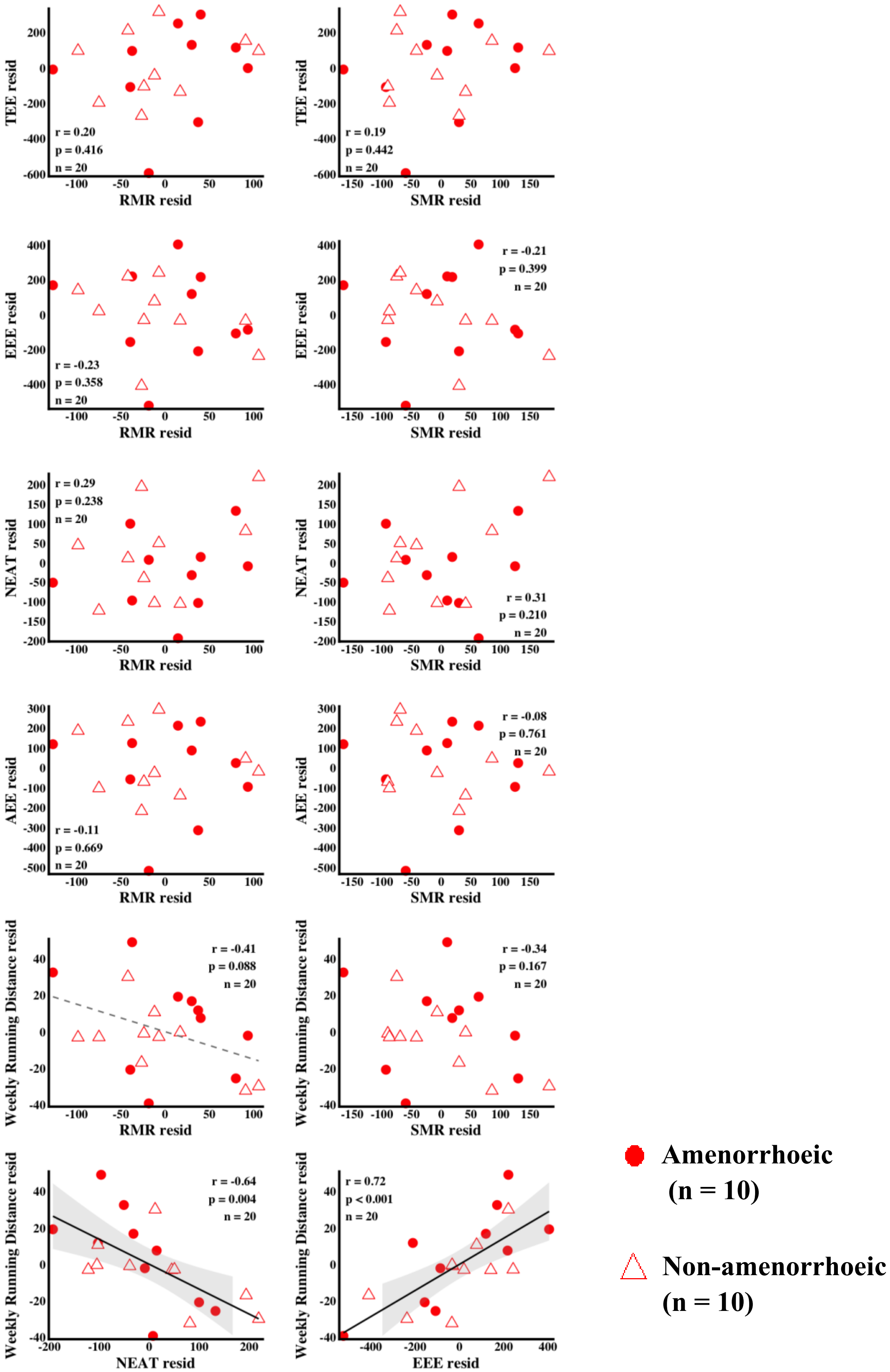

Figure S2. (a) Partial correlations between weekly running distance and energy expenditure components after adjustment for FFM and FM among all female runners (n = 20).

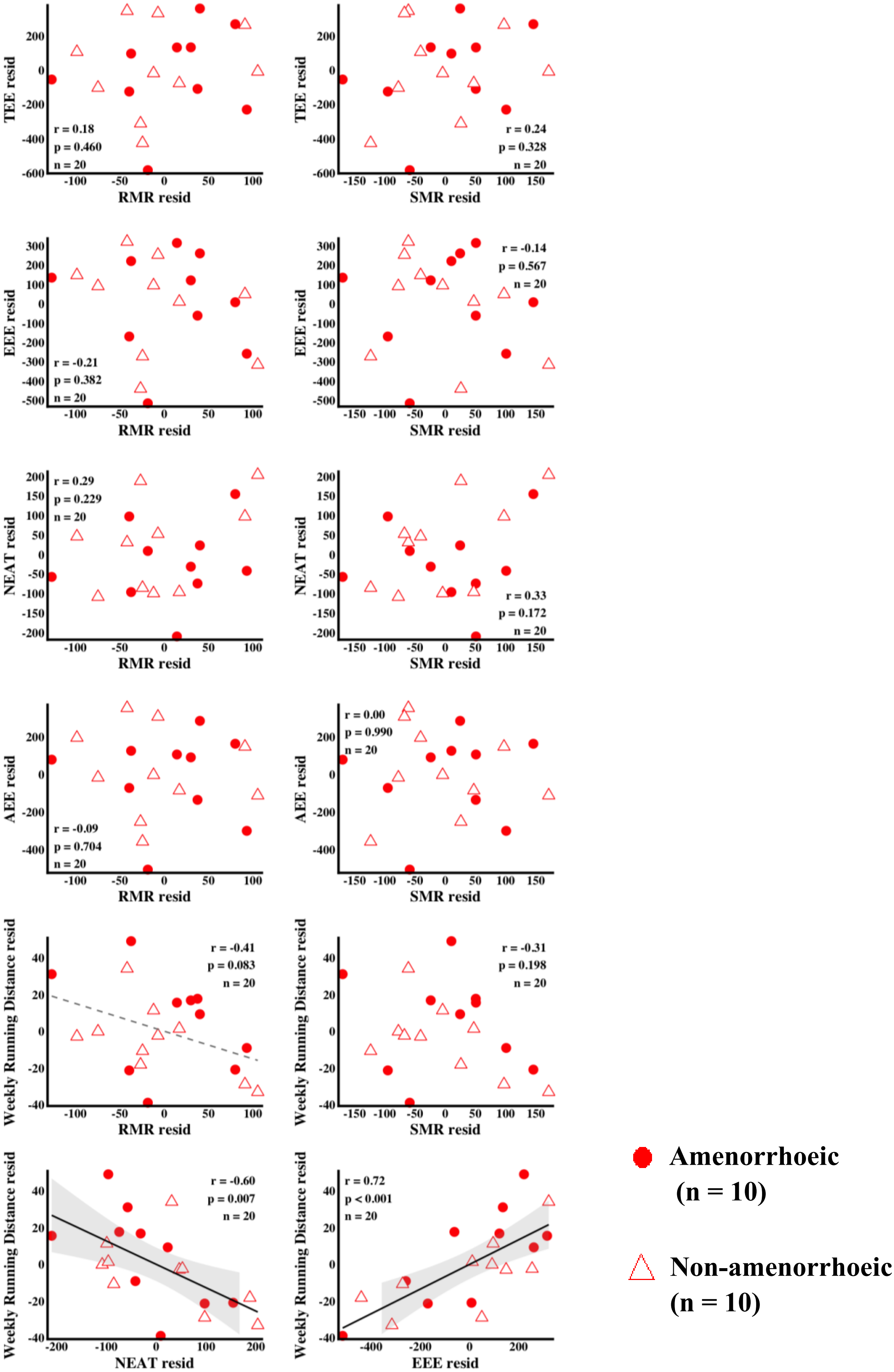

Figure S2. (b) Partial correlations between weekly running distance and energy expenditure components after adjustment for FM among all female runners (n = 20).

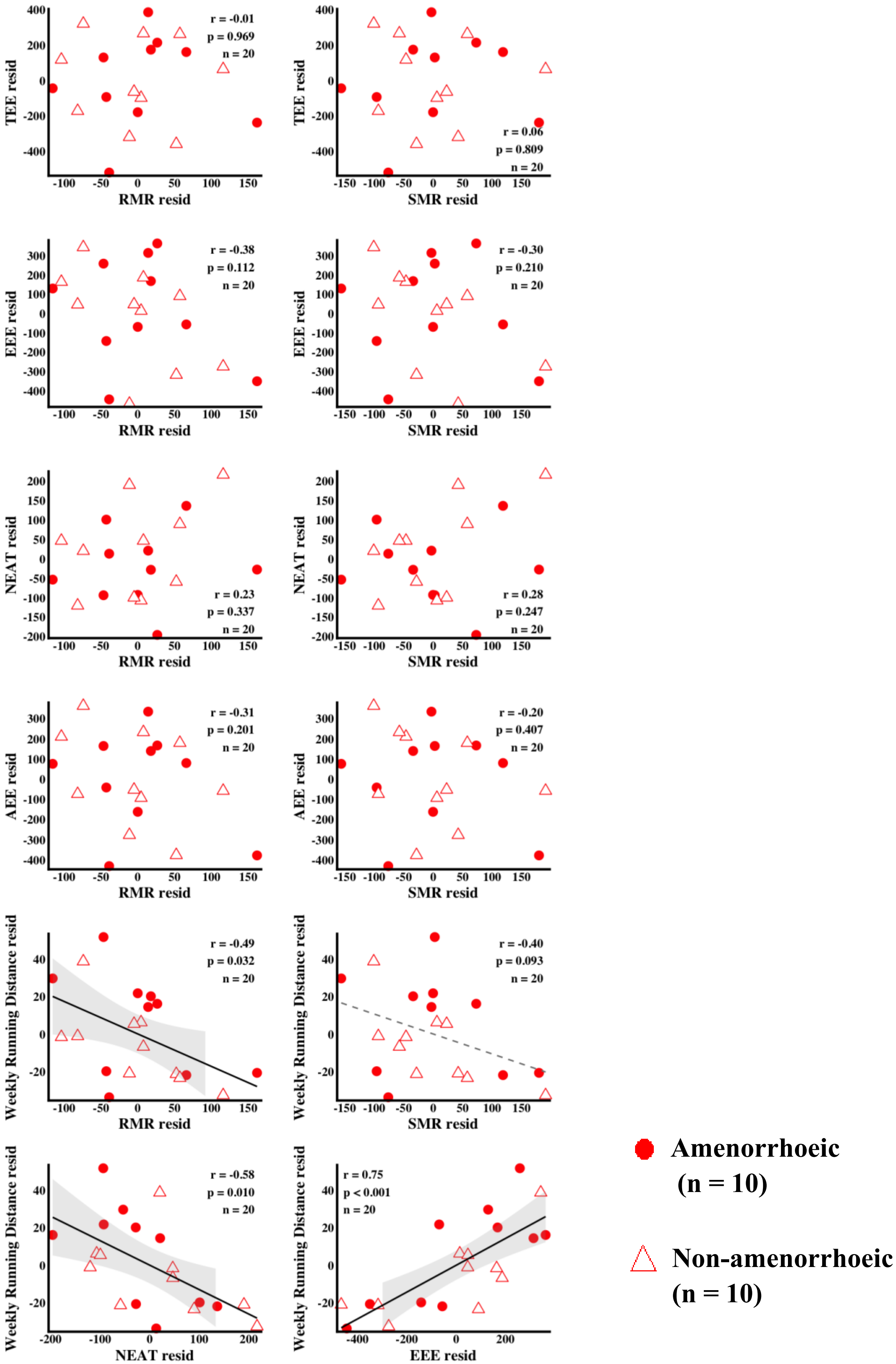

Figure S2. (c) Partial correlations between weekly running distance and energy expenditure components after adjustment for body mass among all female runners (n = 20).
